# Phylogenetic Analysis of Enterovirus 71 Circulating in Beijing, China from 2007 to 2009

**DOI:** 10.1371/journal.pone.0056318

**Published:** 2013-02-13

**Authors:** Junping Zhu, Zhen Luo, Juan Wang, Zigang Xu, Hui Chen, Dongying Fan, Na Gao, Guoling Ping, Zhen Zhou, Yan Zhang, Jing An

**Affiliations:** 1 Department of Pathogenic Biology, School of Basic Medical Sciences, Capital Medical University, Beijing, China; 2 Department of Dermatology, Beijing Children's Hospital, Capital Medical University, Beijing, China; 3 Department of Biomedical Engineering, School of Biomedical Engineering, Capital Medical University, Beijing, China; 4 National Institute for Viral Disease Control and Prevention, Chinese Center for Disease Control and Prevention, Beijing, China; University of Hong Kong, Hong Kong

## Abstract

The major pathogens of hand, foot and mouth disease (HFMD) in Beijing, China from 2007 to 2009 were identified in this study. A total of 186 HFMD cases were included, and 136 cases (73%) were positive for enterovirus (EV). In 2007, 75% (27/36) were Coxsackievirus A16 (CA16) positive and 19% (7/36) were Enterovirus 71 (EV71) positive cases. However, EV71 was the predominant virus in 2008, when 56% (31/55) of the cases were positive for EV71 and 22% (12/55) were positive for CA16. In 2009, EV71 and CA16, with positive rates of 36% (16/45) and 29% (13/45), respectively, were still the major pathogens of HFMD. Phylogenetic analysis revealed that the dominant genotype of EV71 was C4, with co-circulation of genotype A in 2009. The prevalent cluster of the EV71 subgenotype C4 changed over time. A proposed new sublineage of EV71, C4a-2, was the predominant virus associated with the Beijing and nationwide HFMD outbreaks since 2008 and amino acid substitution, which possibly link to the central nervous system tropism of EV71, was found in genotype A viruses. Persistent surveillance of HFMD-associated pathogens is required for predicting potential emerging viruses and related disease outbreaks.

## Introduction

Hand, foot and mouth disease (HFMD) is a common childhood viral infection characterized by mucocutaneous papulovesicular lesions on the hands, feet, mouth and buttocks. The disease is primarily caused by human enterovirus group A (HEV-A) members, which belong to the Picornaviridae family. HEV-A consists of many viruses, including Coxsackievirus A (CA) 2, CA3, CA4, CA5, CA6, CA7, CA8, CA10, CA12, CA14, CA16 and Enterovirus (EV) 71. Of these, EV71 and CA16 are the major etiologic agents of HFMD. In general, typical HFMD cases are mild and self-limited. However, during recent years, it was frequently reported that patients with EV71 infection had severe complications such as acute flaccid paralysis, myocarditis, aseptic meningitis, brainstem encephalitis, neurogenic pulmonary edema and fatal encephalitis. CA16-associated HFMD with severe complications is rarely observed in clinics [1], [2], [3].

EV71 outbreaks have occurred in the Asia-Pacific region and have become an important public health concern since 1997 [4]. The earliest known case of HFMD in China was diagnosed in Shanghai in 1981, and there were subsequently a number of reports of HFMD in most Chinese provinces. A retrospective seroepidemiologic study suggested that EV71 and CA16 had widely circulated in mainland China for a long time [5]. The first HFMD outbreak caused by EV71 was reported in 2007 [6] and it was closely followed by the nationwide HFMD epidemics, which started in Anhui Province in 2008 and subsequently spread quickly to other provinces [7], . Since then there have been annual increases in the number of cases reported, with 1.8 million cases and 905 deaths in 2010 (http://www.chinacdc.cn/tjsj/fdcrbbg/). EV71-associated HFMD has received considerable attention from clinicians and public health officials in China, and HFMD was classified as a category C notifiable infectious disease by the Ministry of Health of China in May 2008. In the Beijing area, a low incidence of HFMD lasted for over 20 years until the nationwide epidemics in 2008, and many HFMD cases have been reported. However, the cause for the sudden national outbreaks remains unclear, and there are currently no effective drugs or vaccines to treat or prevent HFMD.

In the Asia-Pacific region, the dominant EV71 strains circulating in different countries and regions vary genetically, suggesting that different genetic lineages are undergoing rapid evolutionary change [10], [11]. Additionally, almost all outbreaks reported in the Asia-Pacific region during the last decade were caused by previously unidentified EV71 subgenotypes or variants of re-emerging subgenotypes [12]. Accumulating evidence suggested that recombination events could drive the evolution of new genetic lineages [13]. However, in China, nearly all of the related reports have focused on EV71 C4, which is believed to have been circulating predominantly and persistently in China since 1998 [6], [7], [8], [9], [14], [15], [16]. This circulating pattern of unique subgenotype of EV71 C4 differed significantly from the situation of neighboring areas, in which there has been co-circulation or transition of several genotypes during the EV71 epidemics [13].

The aim of this study was to analyze HFMD pathogens circulating in the Beijing area and their genetic features. Clinical specimens were collected from HFMD patients between 2007 and 2009 in Beijing, China, and etiologic agents of the disease were identified by RT-PCR specific to the 5′UTR and a partial region of VP1. Phylogenetic analysis on complete VP1 sequences of the EV71 Beijing strains was performed to investigate their evolution and molecular epidemiology. Our results may provide useful information to explain the sudden outbreak of HFMD and provide insights into disease control.

## Materials and Methods

### Ethical approval

This work was approved by the Ethics Committee of Beijing Children's Hospital, Capital Medical University. The guardians of all the participating children provided written consent for the study.

### RT-PCR assay for the identification of EV71 and CA16 from clinical samples

The samples were collected from the patients on the day of admission to Beijing Children's Hospital, Capital Medical University, and the specimens were stored at −80°C until use. RT-PCR amplification of the highly conserved 5′-untranslated region (UTR) and partial VP1 gene was performed for virus diagnosis.

The clinical specimens were tested by RT-PCR with three primer pairs that screened for panenteroviruses, EV71 and CA16. The highly conserved 5′-UTR of enteroviruses was used in previous study [17] as the target for panenterovirus screening. The primer pair was 5′- CAAGCACTTCTGTTTCCCCGG -3′ and 5′- ATTGTCACCATAAGCAGCCA -3′, which were predicted to amplify a DNA fragment of 435 bp. The primers specific for EV71 and CA16 were used in another previous report [15]. The primers specific for EV71 were 5′- GTCCTTAATTCGCACAGCACAGCT -3′ and 5′- CGGTCCGCACTGAGAATGTACCCAT -3′, which were predicted to amplify a DNA fragment of 507 bp; the primers specific for CA16 were 5′- CCTATTGCAGACATGATTGACCAG -3′ and 5′- TGTTGTTATCTTGTCTCTACTAGTG -3′, which were predicted to amplify a DNA fragment of 881 bp. In brief, viral RNA was extracted from 140 µl samples using the QIAmp® Viral RNA Kit (Qiagen, Hilden, Germany) according to the manufacturer's instructions and transcribed to cDNA with random hexamer primers and the SuperScript II Kit (Invitrogen, USA) at 42°C for 1 h. The PCR amplification was performed in 40 cycles consisting of denaturing for 1 min at 94°C, primer annealing for 1 min at 50°C, and elongation for 1 min at 72°C. The reactions were analyzed by electrophoresis in 1% agarose gels.

To test the detection limit of RT-PCR used in this study, a linearized recombinant plasmid with the specific VP1 sequence of EV71 or CA16 was transcribed to RNA in vitro using the T7 RiboMAXTM Express Large Scale RNA Production System (Promega, USA). The 10-fold serial diluted RNA was used as a template, and the RNA copy number was calculated according to the following formula: number of copies (copies/µl) = amount (µg/µl)×6.022×10^11^/length×340.

To confirm the RT-PCR results, some of samples that tested positive for EV71 or CA16 were further cultured in human rhabdomyosarcoma cells (RD cells, ATCC HTB-139) and examined for the presence of infectious viruses.

### Sequencing the complete VP1 gene of EV71

The primer pair for the complete EV71 VP1 gene was designed in silico using a large sequence database of historical and contemporary viral sequences. The sequences of the primer pair were 5′- GCAGCCCAAAAGAACTTCAC -3′ and 5′- ACCACTCTAAAGTTGCCCAC -3′, which covered the complete VP1 gene and were predicted to amplify a DNA fragment of 1015 bp. The PCR amplification was performed in 34 cycles consisting of denaturing for 30 sec at 94°C, primer annealing for 30 sec at 54°C, and elongation for 1 min 30 sec at 68°C. The PCR products were separated by electrophoresis on a 0.8% agarose gel and subsequently purified using the TaKaRa Agarose Gel DNA Purification Kit (TaKaRa, China). The amplicons were bidirectionally sequenced using an ABI Prism 3730 DNA Analyzer (Applied Biosystems, Foster City, CA, USA) with the appropriate PCR primers by the SinoGenoMax Company (Beijing, China).

### Phylogenetic analysis and nucleotide sequence accession numbers

Sequence alignment of strains was performed with the Clustal W program in MEGA 4 (Molecular Evolutionary Genetics Analysis software; Tamura, Dudley, Nei, and Kumar 2007). Phylogenetic tree based on the complete VP1 gene was constructed using the neighbor joining method. With the MEGA 4.0 software, we implemented the Kimura two-parameter model of nucleotide substitution to calculate genetic distances. Bootstrap analyses were performed on 1,000 replicates to generate confidence for the groupings. The 36 complete VP1 sequences reported in this study were deposited in the GenBank nucleotide sequence database (accession numbers JQ410993 to JQ411010 and JX297492 to JX297509). The reference national and global EV71 strains' sequences used in this study were downloaded from GenBank and were those released before July 16th, 2012. The representative EV71 strains from different regions and different years were selected, and 97 complete VP1 sequences between 1966 and 2011 were obtained. In total, 133 EV71 strains were included in this study, as shown in Table S1 in the supplemental material.

### Analysis of deduced amino acids of VP1 among EV71 genotype A viruses

The complete VP1 sequence alignment of the EV71 genotype A strains was conducted with the Clustal W program in MEGA 4, and then, the deduced amino acid sequences of VP1 protein were aligned. The probable antigenicity of the deduced amino acids in VP1 was predicted using Protean (DNASTAR Lasergene 8.0).

## Results

### General information about our samples and the HFMD cases

HFMD has occurred throughout in the Beijing area each year, and the activity peak of this disease has fallen between April to July in recent years. In this study, a total of 186 HFMD cases were included, and 328 specimens (181 throat swabs and 147 rectal swabs) were collected from outpatients on the day of admission. The percentages of cases collected in this study for the total case number of the outpatients were 18% (51/278) in 2007, 12% (81/657) in 2008 and 12% (54/465) in 2009, respectively. The specimens were collected from April to October from 2007 to 2009. In this study, there was a male predominance in HFMD cases, with a male-to-female ratio of 1.4∶1. The age of the patients ranged from 1 month to 14 years. Of these cases, 88% (163/186) were less than 5 years of age.

### Sensitivity and specificity of our RT-PCR assay

About the sensitivity of RT-PCR assay used in the study, bands of 507 bp (EV71) and 881 bp (CA16) could be observed when the number of RNA copies was not less than 10^2^ copies, indicating that the low limits of detection in our RT-PCR assay were 100 copies/µl for both EV71 and CA16. Furthermore, clinical isolates of EV71, CA16, CA10, CA12, Coxsackievirus B4 (CB4), CB5 and echovirus 30 were used for confirming the specificity of the RT-PCR assay. As expected, a DNA fragment of 507 bp was amplified with the specific primer pair from 5 clinical isolates of EV71, which were negative for the CA16-specific primers. Similarly, a DNA fragment of 881 bp was amplified from 4 CA16 clinical isolates when the specific primer pair for CA16 was used, and all 4 isolates of CA16 were negative for the EV71-specific primers. The other clinical isolates of enteroviruses, including CA10, CA12, CB4, CB5 and echovirus 30, were negative with each primer pair for EV71 and CV16 used in this study, indicating that our RT-PCR assay is specific and can be used in subsequent experiments.

### Changes of the major causative agents of HFMD in the Beijing area from 2007 to 2009

Using our RT-PCR assay, EV was detected in 136 (73%) of 186 cases and 172 (52%) of 328 samples from 2007 to 2009. In 2007, CA16 was the dominant virus, accounting for 75% (27/36) of the laboratory-confirmed EV-positive HFMD cases, whereas EV71 only accounted for 19% (7/36) of the cases. However, in 2008, EV71 became the predominant virus, with a detection rate of 56% (31/55), while CA16 infection was detected in 12 of 55 cases (22%). In 2009, both EV71 and CA16 shared a similar detection rate, with 36% (16/45) and 29% (13/45) for EV71 and CA16, respectively. During those three years, the detection rates of other types of EV infections (excluding EV71 and CA16 infections) also increased from 6% (2/36) in 2007 to 22% (12/55) in 2008 and 16% (7/45) in 2009.

In addition, to further confirm the above results, 37 clinical samples identified as EV71 or CA16 infection were inoculated in RD cells. After three blind passages, all of the cultures exhibited the characteristic EV cytopathic effect, such as cell deformation and necrosis. These results were also validated by RT-PCR and sequencing.

### Genetic characterization of the EV71 strains circulating in Beijing

A total of 133 EV71 strains were used to construct a phylogenetic tree based on the complete VP1 sequences. 36 complete VP1 genes from CMU BJ strains were sequenced in this study. Other sequences were downloaded from GenBank. Thereinto, 20 other BJ strains were from 2006 to 2009, 52 representative strains were detected from more than 19 provinces and 20 cities of mainland China from 1987 to 2011 and 25 international reference strains represented all 12 known EV71 genotypes or subgenotypes (A, B0–B5, C1–C5).

**Figure 1:**
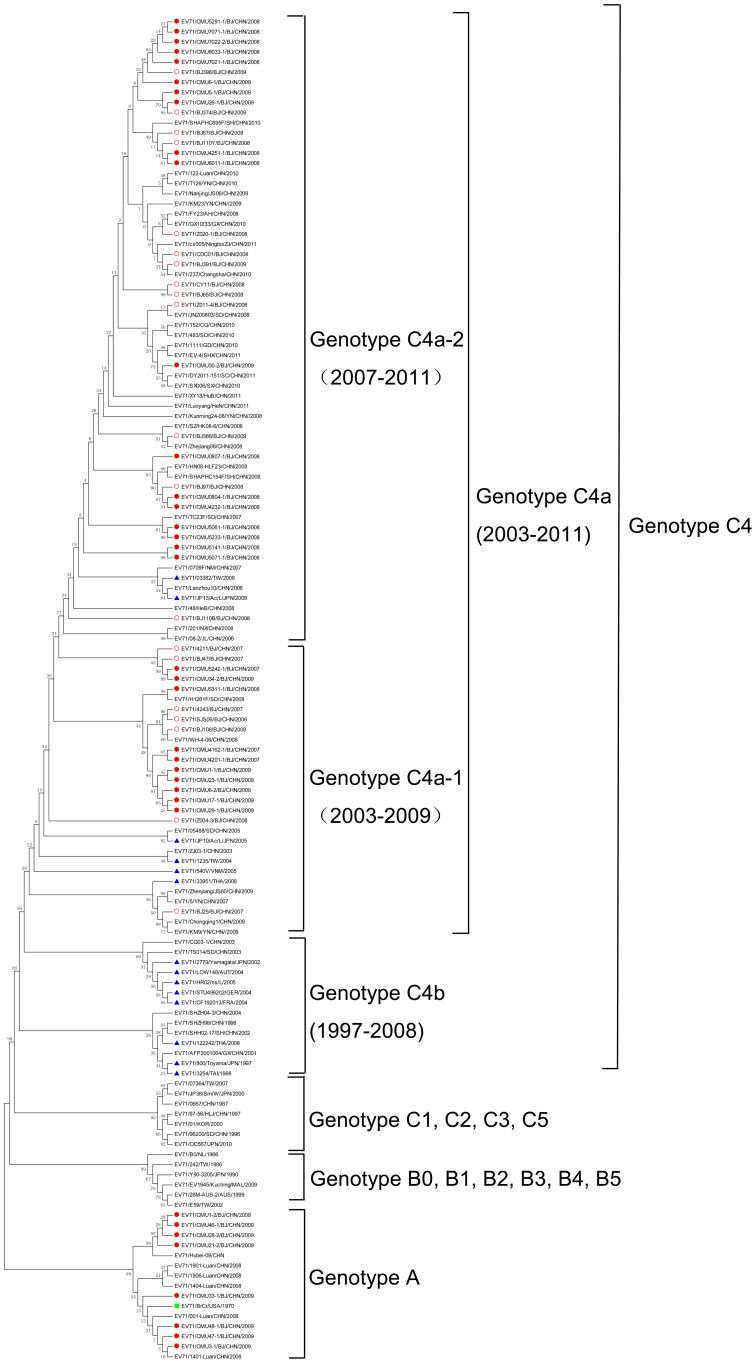
Phylogenetic relationship between 133 worldwide EV71 strains based on the complete VP1 gene. Thirty six CMU BJ strains (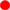), 20 other BJ strains (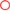), 52 representative CHN strains and 25 international reference strains (including 14 EV71 C4 strains marked with 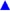, BrCr marked with 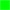 and 10 representative strains of different subgenotypes) are included in this dendrogram. The 52 representative CHN strains were selected from more than 19 provinces and 20 cities in mainland China from 1987 to 2011 according to available locality, time distribution and grouping type. Details of all the EV71 strains included in the dendrogram are provided in Table S1. Country abbreviations: NL, Netherlands; AUS, Australia; KOR, Korea; AUT, Austria; GER, Germany; FRA, France; JPN, Japan; VNM, Vietnam; THA, Thailand; MAL, Malaysia; USA, United States of America; CHN, People's Republic of China. Region abbreviation: TW, Taiwan. Abbreviation of Chinese cities: BJ, Beijing; SH, Shanghai; CQ, Chongqing. Abbreviation of Chinese provinces: HLJ, Heilongjiang; SD, Shandong; GD, Guangdong; GX, Guang Xi; ZJ, Zhejiang; YN, Yunnan; AH, Anhui; HuB, Hubei; JS, Jiangsu; GS, Gansu; HeN, Henan; HeB, Hebei; SC, Si Chuan; SX, Shanxi (taiyuan); SHX, Shanxi (xi'an); NM, Neimeng; NX, Ning Xia; JL, Ji Lin; HuN, Hu Nan.

All of the 133 EV71 strains were separated into three groups as genotypes A, B and C (Figure 1). The analysis revealed that 14 Chinese strains, including 8 CMU BJ strains detected in 2009, 5 strains from Lu'an City in AnHui Province in 2008 and 1 strain from HuBei Province in 2009, were grouped into the genotype A cluster along with the BrCr prototype, and they had a close evolutionary relationship with the BrCr prototype strain (99.0% bootstrap support). In addition, two lineages were identified in this cluster; one contained 4 CMU BJ strains and a Hubei strain, and the other contained the other 4 CMU BJ strains, 5 Luan strains, and the BrCr prototype strain. These CMU BJ EV71 strains of genotype A group appeared to be in two evolutionary lineages, and one lineage might have originated from the BrCr prototype strain. With the exception of several genotype A strains and a few genotype C strains in the early phase, such as EV71/0667/CHN/1987, EV71/96200/SD/CHN/1996 (subgenotype C2) [18] and EV71/97-56/HLJ/CHN/1997 (subgenotype C3), most Chinese EV71 strains (including 28 CMU BJ strains and 20 other BJ strains) were clustered into subgenotype C4, which displayed independent evolution. Subgenotype C4 was further divided into two clusters designated as C4b and C4a. Cluster C4b consisted of strains from 1997 to 2008, including Chinese strains, some European strains from Austria, Germany, France and Croatia, as well as some Southeast Asian strains from Japan, Thailand and Taiwan. Among them, Chinese strains spanned 1998 to 2004. Cluster C4a included strains collected from 2003 to 2011, which were consisted of some Chinese strains and some strains detected from other areas of Southeast Asia, such as Japan, Vietnam, Thailand and Taiwan. Furthermore, there probably are two lineages in cluster C4a. The mean p-distance between these two lineages was 3.9%. Lineage 1 (C4a-1) and 2 (C4a-2) of C4a included strains from 2003–2009 and strains from 2007–2011, respectively. With the exception of the 8 genotype A CMU EV71 strains, all of the remaining 28 CMU strains and 20 other BJ strains detected from 2006 to 2009 belonged to C4a and they also were divided into C4a-1 and C4a-2. All of the C4a-2 BJ strains were collected from 2008 to 2009. This finding indicates that some EV71 strains circulating in the Beijing area since 2008 outbreak tended to be grouped into cluster C4a-2. Similarly, the national strains, including most strains collected in and after 2008 and a few strains detected from Neimeng and Shandong Provinces in 2007, also belonged to C4a-2. Notably, all of the EV71 Chinese strains detected in 2010 and 2011 were clustered into C4a-2. The C4 EV71 cluster showed a step-wise evolutionary trajectory from C4b to C4a-1 to C4a-2. The prevailing cluster changed over time. The mean p-distances within cluster C4a-1 and C4a-2 were 3.5% and 1.8% respectively. EV71 BJ strains were interspersed in the C4a cluster. This indicated that the Beijing strains co-evolved with strains from other regions in mainland China. In general, EV71 in mainland China displayed a national evolution pattern and some circulating EV71 strains had a tendency to group in the new cluster C4a-2 during the large outbreaks that have occurred since 2008.

### Beijing EV71 genotype A-associated HFMD cases and analysis of VP1 amino acid sequences in all genotype A members

The 8 patients with CMU BJ EV71 genotype A infections lived in five different locations. There were 6 males and 2 females, and their ages ranged from 1 year 3 months to 5 years 6 months (median age, 2.88 years). These patients all presented mild symptoms.

The nucleotide identity of the complete VP1 gene among EV71 genotype A strains were 97.2–100%, and the deduced amino acid identities were 94.8–100%. Comparing the deduced amino acid sequences, 26 residue variations were found in the 297 total amino acids of the VP1 protein among EV71 genotype A viruses. The variations in VP1 are listed in Table 1 in three separate sections according to the viral phylogenetic lineage. Of these, 8 mutations, identified as P22Q, P30Q, D31N, R145E, P172Q, S183L, E244K and S246P, were conserved across all genotype A strains, except for BrCr. In addition, some residues (30, 31, 145,183, 244 and 246) were associated with antigenicity, as shown by Protean (Lasergene) predictions.

**Table 1:** Variation of the VP1 amino acid sequences among the EV71 genotype A strains.

EV71 strains	Residue variation in VP1 protein									
	1	2	3	14	18	**22**	**30**	**31**	47	60
EV71/BrCr/USA/1970	G	D	R	D	K	**P**	**P**	**D**	L	D
EV71/CMU33-1/BJ/CHN/2009	-	-	-	-	-	**Q**	**Q**	**N**	-	-
EV71/CMU3-1/BJ/CHN/2009	-	-	-	-	-	**Q**	**Q**	**N**	-	-
EV71/CMU47-1/BJ/CHN/2009	-	-	-	-	-	**Q**	**Q**	**N**	-	-
EV71/CMU48-1/BJ/CHN/2009	-	-	-	-	-	**Q**	**Q**	**N**	-	-
EV71/001-Luan/AH/CHN/2008	-	-	-	-	-	**Q**	**Q**	**N**	-	-
EV71/1401-Luan/AH/CHN/2008	-	G	-	E	-	**Q**	**Q**	**N**	-	-
EV71/1404-Luan/AH/CHN/2008	-	G	-	R	R	**Q**	**Q**	**N**	-	Y
EV71/1901-Luan/AH/CHN/2008	R	G	Q	-	-	**Q**	**Q**	**N**	-	-
E V71/1906-Luan/AH/CHN/2008	-	-	-	-	-	**Q**	**Q**	**N**	-	-
EV71/CMU1-2/BJ/CHN/2009	-	-	-	-	-	**Q**	**Q**	**N**	P	-
EV71/CMU21-2/BJ/CHN/2009	-	-	-	-	-	**Q**	**Q**	**N**	P	-
EV71/CMU28-2/BJ/CHN/2009	-	-	-	-	-	**Q**	**Q**	**N**	P	-
EV71/CMU46-1/BJ/CHN/2009	-	-	-	-	-	**Q**	**Q**	**N**	P	-
EV71/Hubei/HuB/CHN/2009	-	-	-	-	-	**Q**	**Q**	**N**	-	-

	109	111	113	116	117	142	145	164
EV71/BrCr/USA/1970	W	I	I	Y	A	P	**R**	D
EV71/CMU33-1/BJ/CHN/2009	-	-	-	-	-	Y	**E**	-
EV71/CMU3-1/BJ/CHN/2009	-	-	-	-	-	Y	**E**	-
EV71/CMU47-1/BJ/CHN/2009	-	-	-	-	-	Y	**E**	-
EV71/CMU48-1/BJ/CHN/2009	-	-	-	-	-	Y	**E**	-
EV71/001-Luan/AH/CHN/2008	-	-	-	-	-	Y	**E**	-
EV71/1401-Luan/AH/CHN/2008	-	-	-	-	-	Y	**E**	-
EV71/1404-Luan/AH/CHN/2008	-	-	V	-	-	Y	**E**	G
EV71/1901-Luan/AH/CHN/2008	C	-	V	-	-	Y	**E**	G
E V71/1906-Luan/AH/CHN/2008	-	-	V	-	-	Y	**E**	G
EV71/CMU1-2/BJ/CHN/2009	-	V	-	H	V	-	**E**	-
EV71/CMU21-2/BJ/CHN/2009	-	V	-	H	-	-	**E**	-
EV71/CMU28-2/BJ/CHN/2009	-	V	-	H	-	-	**E**	-
EV71/CMU46-1/BJ/CHN/2009	-	V	-	H	-	-	**E**	-
EV71/Hubei/HuB/CHN/2009	-	V	-	H	-	-	**E**	-

	172	183	200	244	246	261	282	297
EV71/BrCr/USA/1970	**P**	**S**	A	**E**	**S**	W	D	L
EV71/CMU33-1/BJ/CHN/2009	**Q**	**L**	-	**K**	**P**	C	-	-
EV71/CMU3-1/BJ/CHN/2009	**Q**	**L**	T	**K**	**P**	-	-	-
EV71/CMU47-1/BJ/CHN/2009	**Q**	**L**	-	**K**	**P**	-	-	-
EV71/CMU48-1/BJ/CHN/2009	**Q**	**L**	-	**K**	**P**	-	-	-
EV71/001-Luan/AH/CHN/2008	**Q**	**L**	-	**K**	**P**	-	-	-
EV71/1401-Luan/AH/CHN/2008	**Q**	**L**	-	**K**	**P**	-	-	-
EV71/1404-Luan/AH/CHN/2008	**Q**	**L**	-	**K**	**P**	-	-	-
EV71/1901-Luan/AH/CHN/2008	**Q**	**L**	-	**K**	**P**	-	-	-
E V71/1906-Luan/AH/CHN/2008	**Q**	**L**	-	**K**	**P**	-	-	-
EV71/CMU1-2/BJ/CHN/2009	**Q**	**L**	-	**K**	**P**	-	N	P
EV71/CMU21-2/BJ/CHN/2009	**Q**	**L**	-	**K**	**P**	-	N	P
EV71/CMU28-2/BJ/CHN/2009	**Q**	**L**	-	**K**	**P**	-	N	P
EV71/CMU46-1/BJ/CHN/2009	**Q**	**L**	-	**K**	**P**	-	N	P
EV71/Hubei/HuB/CHN/2009	**Q**	**L**	-	**K**	**P**	-	-	-

In total, there were 297 amino acids in the VP1 protein of EV71, and 26 residue variations were found in the VP1 amino acid sequence among EV71 genotype A viruses. The 8 amino acid substitutions conserved across all genotype A strains excluding BrCr are marked with bold letters.

Identical = –.

## Discussion

In this study, etiological agents of HFMD in the 2007–2009 epidemic in Beijing were identified. We demonstrated that CA16 was the dominant virus in 2007, while EV71 became the predominant virus in the 2008 outbreak, and both EV71 and CA16 were major pathogens in 2009. Phylogenetic analysis revealed that the dominant genotype of BJ EV71 was C4, with co-circulation of genotype A in 2009. The prevalent cluster of the EV71 subgenotype C4 changed over time. A proposed new sublineage of EV71, C4a-2, was the predominant virus associated with the Beijing and nationwide HFMD outbreaks since 2008.

In agreement with a previous study [19], the global population of EV71 over the past 45 years can be classified into genotypes A, B and C (Figure 1) in this study. Genotypes B and C, which are more commonly reported, consist of subgenogroups B0–B5 and C1–C5, respectively, and the BrCr strain is no longer the sole member of genotype A.

### 1. Emergence of EV71 genotype A

Phylogenetic analysis found that 14 Chinese strains were grouped into one cluster with the BrCr prototype, which was previously thought to be the sole member of genotype A. These Chinese strains were identified in three regions of mainland China, including the Beijing area, the Lu'an city of Anhui Province and its neighbor, HuBei Province. In this study, the EV71 genotype A strains appeared to be separated into two lineages, suggesting that the Chinese EV71 genotype A strains might circulate in mainland China and evolve through two evolutionary chains.

It was reported that a BrCr-associated HFMD patient showed severe neurological symptoms [20]. In contrast, the Beijing HFMD cases caused by EV71 genotype A infection had mild clinical signs, which resembled the symptoms of the patients infected by the Luan strains of EV71 [21]. But the cause associated with change of the viral virulence is not clear and may link to variations in the amino acids of the viral structural proteins.

VP1 is an important protein for viral pathogenicity and is involved in the constitution of the canyon on the surfaces of picornaviruses. Variations in the amino acids of VP1, especially on the inter-surface of the canyon, can likely influence the binding of the virus to its receptor and thus change virus pathogenicity. It has been reported that the D31N mutation might widen the hydrophobic pocket of VP1 to affect VP1-receptor binding [22]. Nishimura et al found that 145E of VP1 might be exposed on the surface of the capsid VP1 protein and could affect receptor binding [23], which has been identified as a major virulence determinant in mouse models [24], [25]. A recent molecular analysis for the virulent determinants of EV71 indicated that 145G/145Q/145R and 164E, located in the DE and EF loops at the surface of the canyon of VP1, could enhance EV71 virulence in humans [26].

Interestingly, 8 mutations found in the VP1 amino acid sequences were conserved across all genotype A strains, except for BrCr, and variations in residues 31D/31N, 145R/145E and 164D/164G were also found in our study. In combination with previous reports, our results may imply that these amino acids (31N, 145E and 164G) of VP1 in all genotype A strains, except for BrCr, might influence the binding ability of virus to the host, and the mutations might link to the central nervous system tropism of EV71. It seems that the amino acids of 31N, 145E and 164G in VP1 are likely associated with virulence of EV71 genotype A strains. However, further investigation is required for clarifying significance of those amino acids in pathogenesis.

### 2. Independent evolution of the EV71 subgenotype C4 and its potential new subdivision

Since 1998, almost all reports of EV71 strains in mainland China have been associated with subgenogroup C4 [6], [7], [8], [9], [14], [15], [16], [27], [28], [29], [30]. This is quite different from that observed in neighboring countries and regions including Malaysia, Singapore, Japan, and Taiwan, where co-circulation and/or transition of different subgenotypes of EV71 strains occurred during the epidemics [2], [11], [31], [32]. In the phylogenetic tree, we observed that the Chinese EV71 subgenotype C4 strains displayed predominant, independent and endemical evolution over the past 14 years. Based on genome analysis, it was found that the EV71 C4 stains circulating in mainland China since 1998 were recombinants of EV71 and CA16 G10 [7]. Moreover, Tee et al proposed that CA16 is the probable ancestor of EV71 [12]. Some recent reports indicated that the Chinese C4 EV71 strains evolve persistently in mainland China since they occurred as a novel subgenotype from around 1992 to 1993 [12], [16]. Our phylogenetic analysis suggested that the Chinese EV71 subgenotype C4 strains evolve endemicity with probable occasional lineage migration among global locations due to travel or business activity. For instance, some EV71 C4 strains were also distributed in European countries in 2004 and 2005 and some Asian countries and regions from 1997 to 2008. The Chinese EV71 strains with the available locality and time distribution exhibited a step-wise evolutionary trajectory through time and the subgenotype C4 EV71 BJ strains did not appear to localize to Beijing; rather, these strains displayed a nationwide pattern, as reported in Guangdong Province [8].

In the phylogenetic tree, the subdivision of subgenotype C4 correlated with the time of virus isolation. C4 viruses displayed 3 phases during their persistent evolution: the early phase, C4b, from 1997 to 2008; the intermediate phase, C4a-1, from 2003 to 2009; and the last phase, C4a-2, from 2007 to 2011. Most members of the C4b group were similar to the C4b subgenotype reported by Zhang [6], while the C4a cluster tended to be further divided into two parts in this study. The results implied that genetic changes persisted during the circulation of subgenotype C4 EV71 strains. In addition to a few strains from the 2007 HFMD Shandong outbreak and the Neimeng epidemic, the proposed C4a-2 lineage mostly consists of strains that were detected during and after the 2008 HFMD national outbreaks, including some BJ strains. This clustering indicated that some EV71 strains circulating in mainland China actually had a tendency to group into a new clade C4a-2. Recent reports revealed that there was exponential growth of the EV71 virus population in 2007 and 2008 in mainland China [16], [33] and the continued replacement of viral lineages occurred through time [33] as we found in this study. We speculate that the potential new cluster transition may have occurred during the 2007 local outbreak in Shandong Province. Subsequently, the viruses of the new C4a-2 strains spread throughout the nation and gradually replaced the intermediate strains of the C4a-1 to become the predominant virus circulating in mainland China since the 2008 national HFMD outbreaks. Previous epidemiology surveys have demonstrated that certain novel EV71 subgenogroups may play a major role in the epidemic. Therefore, we suggest that the potential new evolutionary sublineage C4a-2 might be correlated with the widespread HFMD outbreaks in mainland China that have occurred since 2008. This is supported by a recent study by Tan X., who considered that the most recent descendant from C4a was chiefly responsible for the current large outbreaks [16]. However, further studies are required to validate this potential new subdivision.

### 3. Analysis of causative agents of HFMD in the Beijing area

According to our results, the major pathogen of the Beijing HFMD changed substantially from the 2007 sporadic epidemic to the 2008 outbreak. In 2008, EV71 had a detection rate of 56% compared to the 22% detection rate for CA16. This was consistent with a report [8] in which EV71 (59%) and CA16 (26%) were considered to be the major pathogens involved in the large HFMD outbreak in Guangdong Province of China in 2008. The similar detection rates recorded from Beijing and Guangdong Province indicated that during the 2008 national HFMD outbreak, EV71 may have replaced CA16 to become the major causative agent primarily responsible for the 2008 Beijing HFMD outbreak. In 2009, the EV71 subgenotype C4 co-circulated with the emerging genotype A members in this city, while EV71 and CA16 were still the major pathogens of HFMD. Increases in genetic diversity are correlated with the onset of epidemics [12]. Similar to what was found in other cities in mainland China [6], [7], [28], [34], the co-circulation of other types of EVs and different genotypes of EV71 also recently increased in the Beijing area. The occurrence of genetic recombination is a common event when multiple epidemic strains are circulating simultaneously and the reverse mutations of some amino acid residues in EV71 VP1 protein are also found recently [33]. Therefore, it is necessary to pay attention to the causative agents that co-circulate during HFMD outbreaks to track the potential emergence and spread of novel recombinants or reverse mutants and the probable related epidemics.

## Supporting Information

Table S1 **The list of EV71 strains used for analysis in this study.**
(DOC)
