# Supplementary material for: Phylogenetic Analysis of Enterovirus 71 Circulating in Beijing, China from 2007 to 2009
Source: PLoS One. 2013 Feb 13;8(2):e56318. doi: 10.1371/journal.pone.0056318 (PMC3572022; doi:10.1371/journal.pone.0056318)
Supplement: Table S1 — The list of EV71 strains used for analysis in this study. (DOC) [file pone.0056318.s001.doc]

**Table S1．The list of EV71 strains used for analysis** in this study.

| Strain | Year of isolation | Place of isolation | Genogroup | GenBank no. | Source |
| --- | --- | --- | --- | --- | --- |
| EV71/CMU4201-1/BJ/CHN/2007 | 2007 | BJ/CHN | C4a-1 | JX297492 | this study |
| EV71/CMU4162-1/BJ/CHN/2007 | 2007 | BJ/CHN | C4a-1 | JX297493 | this study |
| EV71/CMU5242-1/BJ/CHN/2007 | 2007 | BJ/CHN | C4a-1 | JX297494 | this study |
| EV71/CMU0804-1/BJ/CHN/2008 | 2008 | BJ/CHN | C4a-2 | JX297495 | this study |
| EV71/CMU0807-1/BJ/CHN/2008 | 2008 | BJ/CHN | C4a-2 | JX297496 | this study |
| EV71/CMU4232-1/BJ/CHN/2008 | 2008 | BJ/CHN | C4a-2 | JX297497 | this study |
| EV71/CMU4251-1/BJ/CHN/2008 | 2008 | BJ/CHN | C4a-2 | JX297498 | this study |
| EV71/CMU5061-1/BJ/CHN/2008 | 2008 | BJ/CHN | C4a-2 | JX297499 | this study |
| EV71/CMU5071-1/BJ/CHN/2008 | 2008 | BJ/CHN | C4a-2 | JX297500 | this study |
| EV71/CMU5141-1/BJ/CHN/2008 | 2008 | BJ/CHN | C4a-2 | JX297501 | this study |
| EV71/CMU5233-1/BJ/CHN/2008 | 2008 | BJ/CHN | C4a-2 | JX297502 | this study |
| EV71/CMU5291-1/BJ/CHN/2008 | 2008 | BJ/CHN | C4a-2 | JX297503 | this study |
| EV71/CMU5311-1/BJ/CHN/2008 | 2008 | BJ/CHN | C4a-1 | JX297504 | this study |
| EV71/CMU6011-1/BJ/CHN/2008 | 2008 | BJ/CHN | C4a-2 | JX297505 | this study |
| EV71/CMU7021-1/BJ/CHN/2008 | 2008 | BJ/CHN | C4a-2 | JX297506 | this study |
| EV71/CMU7022-2/BJ/CHN/2008 | 2008 | BJ/CHN | C4a-2 | JX297507 | this study |
| EV71/CMU7071-1/BJ/CHN/2008 | 2008 | BJ/CHN | C4a-2 | JX297508 | this study |
| EV71/CMU8033-1/BJ/CHN/2008 | 2008 | BJ/CHN | C4a-2 | JX297509 | this study |
| EV71/CMU1-1/BJ/CHN/2009 | 2009 | BJ/CHN | C4a-1 | JQ410993 | this study |
| EV71/CMU1-2/BJ/CHN/2009 | 2009 | BJ/CHN | A | JQ410994 | this study |
| EV71/CMU3-1/BJ/CHN/2009 | 2009 | BJ/CHN | A | JQ410995 | this study |
| EV71/CMU5-1/BJ/CHN/2009 | 2009 | BJ/CHN | C4a-2 | JQ410996 | this study |
| EV71/CMU6-1/BJ/CHN/2009 | 2009 | BJ/CHN | C4a-2 | JQ410997 | this study |
| EV71/CMU6-2/BJ/CHN/2009 | 2009 | BJ/CHN | C4a-1 | JQ410998 | this study |
| EV71/CMU17-1/BJ/CHN/2009 | 2009 | BJ/CHN | C4a-1 | JQ410999 | this study |
| EV71/CMU21-2/BJ/CHN/2009 | 2009 | BJ/CHN | A | JQ411000 | this study |
| EV71/CMU23-1/BJ/CHN/2009 | 2009 | BJ/CHN | C4a-1 | JQ411001 | this study |
| EV71/CMU26-1/BJ/CHN/2009 | 2009 | BJ/CHN | C4a-2 | JQ411002 | this study |
| EV71/CMU28-2/BJ/CHN/2009 | 2009 | BJ/CHN | A | JQ411003 | this study |
| EV71/CMU29-1/BJ/CHN/2009 | 2009 | BJ/CHN | C4a-1 | JQ411004 | this study |
| EV71/CMU30-2/BJ/CHN/2009 | 2009 | BJ/CHN | C4a-2 | JQ411005 | this study |
| EV71/CMU33-1/BJ/CHN/2009 | 2009 | BJ/CHN | A | JQ411006 | this study |
| EV71/CMU34-2/BJ/CHN/2009 | 2009 | BJ/CHN | C4a-1 | JQ411007 | this study |
| EV71/CMU46-1/BJ/CHN/2009 | 2009 | BJ/CHN | A | JQ411008 | this study |
| EV71/CMU47-1/BJ/CHN/2009 | 2009 | BJ/CHN | A | JQ411009 | this study |
| EV71/CMU48-1/BJ/CHN/2009 | 2009 | BJ/CHN | A | JQ411010 | this study |
| EV71/SJS06/BJ/CHN/2006 | 2006 | BJ/CHN | C4a-1 | HQ129932 | GenBank |
| EV71/4211/BJ/CHN/2007 | 2007 | BJ/CHN | C4a-1 | EU024958 | GenBank |
| EV71/4243/BJ/CHN/2007 | 2007 | BJ/CHN | C4a-1 | EU019910 | GenBank |
| EV71/BJ47/BJ/CHN/2007 | 2007 | BJ/CHN | C4a-1 | JF317976 | GenBank |
| EV71/BJ25/BJ/CHN/2007 | 2007 | BJ/CHN | C4a-1 | JF317975 | GenBank |
| EV71/CY11/BJ/CHN/2008 | 2008 | BJ/CHN | C4a-2 | FJ469153 | GenBank |
| EV71/CDC01/BJ/CHN/2008 | 2008 | BJ/CHN | C4a-2 | FJ765424 | GenBank |
| EV71/Z004-3/BJ/CHN/2008 | 2008 | BJ/CHN | C4a-1 | FJ606447 | GenBank |
| EV71/Z020-1/BJ/CHN/2008 | 2008 | BJ/CHN | C4a-2 | FJ606449 | GenBank |
| EV71/Z011-4/BJ/CHN/2008 | 2008 | BJ/CHN | C4a-2 | FJ606448 | GenBank |
| EV71/BJ65/BJ/CHN/2008 | 2008 | BJ/CHN | C4a-2 | JF317977 | GenBank |
| EV71/BJ67/BJ/CHN/2008 | 2008 | BJ/CHN | C4a-2 | JF317978 | GenBank |
| EV71/BJ108/BJ/CHN/2008 | 2008 | BJ/CHN | C4a-1 | JF317980 | GenBank |
| EV71/BJ110Y/BJ/CHN/2008 | 2008 | BJ/CHN | C4a-2 | JF317982 | GenBank |
| EV71/BJ110B/BJ/CHN/2008 | 2008 | BJ/CHN | C4a-2 | JF317981 | GenBank |
| EV71/BJ97/BJ/CHN/2008 | 2008 | BJ/CHN | C4a-2 | JF317979 | GenBank |
| EV71/BJ398/BJ/CHN/2009 | 2009 | BJ/CHN | C4a-2 | JF317986 | GenBank |
| EV71/BJ374/BJ/CHN/2009 | 2009 | BJ/CHN | C4a-2 | JF317984 | GenBank |
| EV71/BJ391/BJ/CHN/2009 | 2009 | BJ/CHN | C4a-2 | JF317985 | GenBank |
| EV71/BJ366/BJ/CHN/2009 | 2009 | BJ/CHN | C4a-2 | JF317983 | GenBank |
| EV71/0667/CHN/1987 | 1987 | CHN | / | AF135934 | GenBank |
| EV71/96200/SD/CHN/1996 | 1996 | SD/ CHN | C2 | JQ326306 | GenBank |
| EV71/97-56/HLJ/CHN/1997 | 1997 | HLJ/CHN | C3 | AB115494 | GenBank |
| EV71/TS014/SD/CHN/2003 | 2003 | SD/ CHN | C4b | HM212443 | GenBank |
| EV71/05488/SD/CHN/2005 | 2005 | SD/ CHN | C4a-1 | GQ253421 | GenBank |
| EV71/TC23F/SD/CHN/2007 | 2007 | SD/ CHN | C4a-2 | EU753417 | GenBank |
| EV71/H1261F/SD/CHN/2008 | 2008 | SD/ CHN | C4a-1 | GQ253399 | GenBank |
| EV71/JN200803/SD/CHN/2008 | 2008 | SD/ CHN | C4a-2 | JF913464 | GenBank |
| EV71/483/SD/CHN/2010 | 2010 | SD/ CHN | C4a-2 | HQ668421 | GenBank |
| EV71/SHZH98/CHN/1998 | 1998 | GD /CHN | C4b | AF302996 | GenBank |
| EV71/SHZH04-3/CHN/2004 | 2004 | GD /CHN | C4b | AY895142 | GenBank |
| EV71/SZ/HK08-6/CHN/2008 | 2008 | GD/CHN | C4a-2 | GQ279370 | GenBank |
| EV71/1111/GD/CHN/2010 | 2010 | GD/ CHN | C4a-2 | JF519718 | GenBank |
| EV71/SHH02-17/SH/CHN/2002 | 2002 | SH/CHN | C4b | AY547500 | GenBank |
| EV71/SHAPHC154F/SH/CHN/2008 | 2008 | SH/CHN | C4a-2 | HM579945 | GenBank |
| EV71/SHAPHC695F/SH/CHN/2010 | 2010 | SH/CHN | C4a-2 | JQ736684 | GenBank |
| EV71/AFP2001064/GX/CHN/2001 | 2001 | GX/CHN | C4b | JQ742001 | GenBank |
| EV71/GX10/33/GX/CHN/2010 | 2010 | GX/CHN | C4a-2 | HQ428125 | GenBank |
| EV71/CQ03-1/CHN/2003 | 2003 | CQ/ CHN | C4b | AY547501 | GenBank |
| EV71/Chongqing1/CHN/2009 | 2009 | CQ/ CHN | C4a-1 | GQ994989 | GenBank |
| EV71/152/CQ/CHN/2010 | 2010 | CQ/ CHN | C4a-2 | HQ668439 | GenBank |
| EV71/ZJ03-1/CHN/2003 | 2003 | ZJ/ CHN | C4a-1 | AY905614 | GenBank |
| EV71/Zhejiang08/CHN/2008 | 2008 | ZJ/ CHN | C4a-2 | EU864507 | GenBank |
| EV71/cx005/Ningbo/ZJ/CHN/2011 | 2011 | ZJ/ CHN | C4a-2 | JQ284022 | GenBank |
| EV71/5/YN/CHN/2007 | 2007 | YN/ CHN | C4a-1 | AB725672 | GenBank |
| EV71/Kunming24-08/YN/CHN//2008 | 2008 | YN/ CHN | C4a-2 | FJ765425 | GenBank |
| EV71/KM9/YN/CHN//2009 | 2009 | YN/ CHN | C4a-1 | JF505389 | GenBank |
| EV71/KM23/YN/CHN//2009 | 2009 | YN/ CHN | C4a-2 | JF505392 | GenBank |
| EV71/T126/YN/CHN/2010 | 2010 | YN/ CHN | C4a-2 | JN256068 | GenBank |
| EV71/1901-Luan/CHN/2008 | 2008 | AH/CHN | A | GQ117127 | GenBank |
| EV71/001-Luan/CHN/2008 | 2008 | AH/CHN | A | GQ117124 | GenBank |
| EV71/1906-Luan/CHN/2008 | 2008 | AH/CHN | A | GQ117128 | GenBank |
| EV71/1404-Luan/CHN/2008 | 2008 | AH/CHN | A | GQ117126 | GenBank |
| EV71/1401-Luan/CHN/2008 | 2008 | AH/CHN | A | GQ117125 | GenBank |
| EV71/FY23/AH/CHN/2008 | 2008 | AH/ CHN | C4a-2 | EU812515 | GenBank |
| EV71/122-Luan/CHN/2010 | 2010 | AH/CHN | C4a-2 | JF937668 | GenBank |
| EV71/WH-4-08/CHN/2008 | 2008 | HuB/ CHN | C4a-1 | FJ765433 | GenBank |
| EV71/Hubei-09/CHN/2009 | 2009 | HuB/CHN | A | GU434678 | GenBank |
| EV71/XY18/HuB/CHN/2011 | 2011 | HuB/CHN | C4a-2 | JQ906805 | GenBank |
| EV71/Zhenjiang/JS60/CHN/2009 | 2009 | JS/CHN | C4a-1 | GU353106 | GenBank |
| EV71/Nanjing/JS06/CHN/2009 | 2009 | JS/CHN | C4a-2 | GU353080 | GenBank |
| EV71/Lanzhou10/CHN/2008 | 2008 | GS/CHN | C4a-2 | GQ855294 | GenBank |
| EV71/HN08-HLF23/CHN/2008 | 2008 | HeN/CHN | C4a-2 | HM038015 | GenBank |
| EV71/Luoyang/HeN/CHN/2011 | 2011 | HeN/CHN | C4a-2 | JN020147 | GenBank |
| EV71/48/HeB/CHN/2008 | 2008 | HeB/CHN | C4a-2 | HM212449 | GenBank |
| EV71/DY2011-151/SC/CHN/2011 | 2011 | SC/CHN | C4a-2 | AB675651 | GenBank |
| EV71/SX006/SX/CHN/2010 | 2010 | SX(taiyuan) /CHN | C4a-2 | JN256067 | GenBank |
| EV71/EV-4/SHX/CHN/2011 | 2011 | SHX (xi’an)/CHN | C4a-2 | JN712916 | GenBank |
| EV71/0709F/NM/CHN/2007 | 2007 | NM/ CHN | C4a-2 | EU910862 | GenBank |
| EV71/201/NX/CHN/2008 | 2008 | NX/CHN | C4a-2 | HM212463 | GenBank |
| EV71/08-2/JL/CHN/2008 | 2008 | JL/CHN | C4a-2 | HM212452 | GenBank |
| EV71/237/Changsha/CHN/2010 | 2010 | HuN/CHN | C4a-2 | HM776019 | GenBank |
| EV71/B0/NL/1966 | 1966 | NL | B0 | AB552973 | GenBank |
| EV71/26M-AUS-2/AUS/1999 | 1999 | AUS | B3 | AF376101 | GenBank |
| EV71/01/KOR/2000 | 2000 | KOR | C3 | AY125966 | GenBank |
| EV71/HR02/ns/L/2005 | 2005 | Croatia | C4b | HQ676186 | GenBank |
| EV71/LOW148/AUT/2004 | 2004 | AUT | C4b | FN649269 | GenBank |
| EV71/STU499202/GER/2004 | 2004 | GER | C4b | FN649254 | GenBank |
| EV71/CF192013/FRA/2004 | 2004 | FRA | C4b | FN598741 | GenBank |
| EV71/242/TW/1986 | 1986 | TW | B1 | JN874548 | GenBank |
| EV71/3254/TAI/1998 | 1998 | TW | C4b | AF286531 | GenBank |
| EV71/E59/TW/2002 | 2002 | TW | B4 | JN874551 | GenBank |
| EV71/1235/TW/2004 | 2004 | TW | C4a-1 | DQ133459 | GenBank |
| EV71/07364/TW/2007 | 2007 | TW | C5 | EU527983 | GenBank |
| EV71/03382/TW/2008 | 2008 | TW | C4a-2 | JF420547 | GenBank |
| EV71/Y90-3205/JPN/1990 | 1990 | JPN | B2 | AB433863 | GenBank |
| EV71/800/Toyama/JPN/1997 | 1997 | JPN | C4b | AB465410 | GenBank |
| EV71/JP53/Yg/W/JPN/1998 | 1998 | JPN | C2 | HQ676232 | GenBank |
| EV71/JP38/Sm/W/JPN/2000 | 2000 | JPN | C1 | HQ676217 | GenBank |
| EV71/2779/Yamagata/JPN/2002 | 2002 | JPN | C4b | AB213629 | GenBank |
| EV71/JP10/Ac/L/JPN/2005 | 2005 | JPN | C4a-1 | HQ676197 | GenBank |
| EV71/JP13/Ac/L/JPN/2008 | 2008 | JPN | C4a-2 | HQ676200 | GenBank |
| EV71/540V/VNM/2005 | 2005 | VNM | C4a-1 | AM490151 | GenBank |
| EV71/12242/THA/2008 | 2008 | THA | C4b | FJ151494 | GenBank |
| EV71/33951/THA/2008 | 2008 | THA | C4a-1 | FJ556875 | GenBank |
| EV71/EV1945/Kuching/MAL/2009 | 2009 | MAL | B5 | HM358835 | GenBank |
| EV71/BrCr/USA/1970 | 1970 | USA | A | U22521 | GenBank |

There are 133 complete VP1 sequences of EV71 strains were involved in this study totally. The EV71 strains from different regions of mainland China, were selected according to available time distribution and in different groupings.

Abbreviation of Chinese cities: BJ, Beijing；SH, Shanghai; CQ, Chongqing. Abbreviation of Chinese provinces: HLJ, Heilongjiang ; SD, Shandong; GD, Guangdong; GX, Guang Xi; ZJ, Zhejiang; YN, Yunnan; AH, Anhui; HuB, Hubei; JS, Jiangsu; GS, Gansu; HeN, Henan; HeB, Hebei; SC, Si Chuan; SX, Shanxi(taiyuan); SHX, Shanxi (xi’an); NM, Neimeng; NX, Ning Xia; JL, Ji Lin; HuN, Hu Nan.

Region abbreviation: TW, Taiwan.

Country abbreviations: NL, Netherlands; AUS, Australia; KOR, Korea; AUT, Austria; GER, Germany; FRA, France; JPN, Japan; VNM, [Vietnam](app:ds:Vietnam); THA, Thailand; MAL, Malaysia; USA, United States of America; CHN, People’s Republic of China.

C4a-1: lineage 1 of subgenotype C4a; C4a-2: lineage 2 of subgenotype C4a.
